# AGE DIFFERENCES IN COGNITIVE PERSONAL NETWORKS

**DOI:** 10.1093/geroni/igz038.629

**Published:** 2019-11-08

**Authors:** Christopher S Marcum, Jielu Lin, Laura M Koehly

**Affiliations:** 1 National Institutes of Health, Bethesda, Maryland, United States; 2 Northern Arizona University, Flagstaff, Arizona, United States; 3 National Human Genome Research Institute, National Institutes of Health, Bethesda, Maryland, United States

## Abstract

Previous research has found a negative association between network size and age, suggesting that people experience greater isolation with advancing age. In this paper, we evaluate age differences in how individuals perceive their social worlds to be structured, rather than focusing solely on network size. A nationally represented sample of respondents (n=1,824) reported on their own ties to their close personal network members (i.e., ego-alter ties) as well as their perceptions of acquaintanceship between those members (i.e., alter-alter ties). We used social network analysis to assess how the structure of these relationships vary by respondent age. We find a positive association between respondent age and personal network size and a negative association between network members’ ages and the number of ties respondents’ perceive their members to have to each other. This effect significantly weakens as respondent age increases. Moreover, we find evidence of age-homophily, intergenerational contact spanning three generations in both ego-alter and alter-alter ties, and age differences in ego network composition. Our results suggest that the evolution of our social worlds across the life course shifts in terms of size and structure. While contemporary close personal networks may grow slightly with age, perceived social ties among one’s network members become less cohesive and less diverse with age. We discuss these results in the context of recent findings that suggest aging uniformly insulates individuals from social contact from both structural and symbolic perspectives.

